# Updates in Neuropsychiatric Systemic Lupus Erythematosus: Advances in Pathogenesis, Diagnosis, and Therapies

**DOI:** 10.7759/cureus.117661

**Published:** 2026-10-08

**Authors:** Dania El Natour, Yahya Makkieh, Taymour El Shaar, Hadi El Natour

**Affiliations:** 1 Department of Family Medicine, American University of Beirut Medical Center, Beirut, LBN; 2 Faculty of Medicine, Beirut Arab University, Beirut, LBN

**Keywords:** autoantibodies, autoimmune diseases, bbb permeability, neuropsychiatric systemic lupus erythematosus (npsle), personalized medicine

## Abstract

Neuropsychiatric systemic lupus erythematosus (NPSLE) encompasses a diverse range of neurological and psychiatric manifestations occurring in patients with systemic lupus erythematosus (SLE); however, these events are ultimately attributed to NPSLE only after rigorous exclusion of alternative etiologies and secondary causes. These manifestations result directly from lupus-related inflammation, vascular damage, or autoantibody effects on the central and peripheral nervous systems. The purpose of this review is to provide an updated overview of NPSLE, the multidisciplinary approach in diagnosing NPSLE, the different proposed mechanisms of pathophysiology, as well as the available treatment options. It also sheds light on the introduced advancements in personalized targeted therapy for NPSLE.

A comprehensive review of the relevant literature on the state of NPSLE diagnosis, pathogenesis, and treatment was conducted. Different search databases (PubMed, Google Scholar, ScienceDirect) were searched for studies published, prioritizing the most recent ones. Search terms included "neuropsychiatric manifestations of SLE", "diagnostic biomarkers", "targeted therapy", and "autoantibodies and neurotoxicity". Only English-language articles were included. As a narrative review, the selection was not systematic and may be subject to selection bias.

The review presented the most evident recent diagnostic approaches of NPSLE, including those currently in clinical use and those still under investigation. Moreover, the complex pathogenesis of autoantibodies, inflammatory cells, and cytokines, along with other environmental and genetic factors, was discussed. The emerging and promising strategies for NPSLE management targeting proposed or emerging pathogenic pathways offer new opportunities to treat one of the most severe complications of SLE.

The growing understanding of the pathogenesis of NPSLE paves the way for targeted therapy and symptom management. Despite promising advances, actual clinical trials are needed to evaluate the efficacy and safety of the proposed treatment strategies. Future research should focus on validating the personalized targeted interventions and ultimately improving prognosis and outcomes for patients suffering from this severe complication of NPSLE.

## Introduction and background

Systemic lupus erythematosus (SLE) is an autoimmune disease that can affect almost any organ system. It is characterized by immune reactivity against multiple nuclear components that leads to damage to different organs including the skin, central nervous system (CNS), joints, kidneys, and heart [1]. Currently, around 3.41 million people are diagnosed with SLE with a global increase in prevalence due to multiple underlying factors [2]. This burden is accompanied by higher mortality rates, particularly among females, younger individuals, those with shorter disease duration, and Black/African American patients [3]. Among the hallmarks of SLE pathogenesis is immune dysregulation, intricately due to multifactorial interactions including genetic predisposition, hormonal imbalances, and environmental influences [4]. SLE is commonly known for its highly heterogeneous clinical presentation, manifesting with diverse and often unpredictable phenotypes. Its symptoms can vary from a very mild disease to life-threatening conditions [5].

Neuropsychiatric systemic lupus erythematosus (NPSLE) is one of its many forms that presents as a complex condition with significant diagnostic importance. It is now the focus of extensive research aimed at improving diagnosis, understanding its pathophysiology, and developing more effective, targeted therapies. It is a clinically significant manifestation of SLE with a reported prevalence of 44.5% in prospective studies, compared to 17.6% in retrospective analyses [6]. This discrepancy is largely attributed to study design; prospective studies utilize standardized, active screening protocols that capture milder or transient neuropsychiatric symptoms, whereas retrospective analyses rely on medical records that typically only document more severe or overt clinical events. It plays a significant role in prognosis as it is linked to impaired functional outcomes, a decline in quality of life over time, and increased mortality [7].

The American College of Rheumatology (ACR) utilizes overlapping descriptive frameworks to classify NPSLE manifestations, categorizing them into CNS and peripheral nervous system (PNS) syndromes, which can concurrently be described as either focal or diffuse [8]. Moreover, they can also be categorized based on their pathophysiological processes, as inflammatory or thrombotic/ischemic. "Secondary NPSLE" describes neuropsychiatric symptoms arising from SLE treatment or SLE-related organ damage [9].

NPSLE's most common clinical manifestations include headache, mood disorders, seizures, cognitive dysfunction, and cerebrovascular disease. Other less reported ones include plexopathy, Guillain-Barré syndrome, myasthenia gravis, and autonomic dysfunction [6]. This heterogeneity in presentations highlights the multifactorial nature of NPSLE and the importance of thorough clinical evaluation.

Current research focuses on improving the accuracy of NPSLE diagnosis and management. Diagnosis typically involves a comprehensive approach focused on excluding NPSLE mimics, like infections, metabolic disturbances, and drug-induced effects [10]. Diagnosis remains primarily a clinical process of attribution after excluding alternative causes. While magnetic resonance imaging (MRI) and testing for established autoantibodies like antiphospholipid antibodies (aPL) are used routinely in clinical practice, other circulating autoantibodies, such as anti-NR2A/2B (against the NR2A and NR2B subunits of the N-methyl-D-aspartate (NMDA) receptors), anti-MAP-2 (against the microtubule-associated protein 2), and anti-Rib-P (against ribosomal phosphoproteins), are largely investigational and considered candidate research biomarkers rather than standard diagnostic tools. In addition, the cerebrospinal fluid (CSF) analysis, electrophysiological studies, neuropsychological assessments, and imaging modalities, such as computed tomography (CT), positron emission tomography (PET), and magnetic resonance angiography (MRA), aid in diagnosis to guide the appropriate therapeutic interventions [11,12].

Although acute stress enhances immunity transiently, chronic stress disturbs homeostasis, elevating pro-inflammatory cytokines, such as IL-1β, IL-6, and TNF-α, and often causing glucocorticoid resistance, which contributes to the ongoing neuroinflammation. Drawing from broader neuroinflammatory literature and preclinical models, it is hypothesized that these circulating cytokines may cross the blood-brain barrier (BBB), activating microglia and altering neurotransmission, which could contribute to neuropsychological symptoms like mood disturbances, cognitive deficits, and fatigue [13]. In autoimmune conditions such as NPSLE, this proposed stress-immune-brain loop is thought to disrupt CNS homeostasis and may underlie certain neuropsychiatric symptoms [14]. A deeper understanding of these theoretical mechanisms is crucial for guiding future research toward targeted diagnostics and therapeutics.

Nowadays, the neuroimmune basis of NPSLE is increasingly recognized. However, there remain key gaps regarding the mechanism of cytokine activation, the causal pathways, and, more importantly, finding reliable biomarkers for diagnosis and treatment monitoring. Currently, while available therapeutic strategies target broad immune pathways, they largely lack precise neuroimmune specificity and often fail to fully address the profound clinical heterogeneity of NPSLE [10]. Because NPSLE is not a single pathophysiological entity, distinguishing between inflammatory and thrombotic/ischemic mechanisms, and accurately attributing these events to SLE, is fundamental to interpreting the utility of emerging biomarkers and targeted therapies. This review addresses in depth the neuropsychiatric manifestations and the most studied diagnostic approaches and synthesizes current evidence on CNS-immune interactions in NPSLE. Moreover, it explores emerging directions in personalized immunotherapy and highlights the need for more research and trials on the proposed targeted approach to diagnosis and management by integrating multidisciplinary insights.

## Review

NPSLE

ACR defines NPSLE as one or more of 19 distinct neuropsychiatric syndromes involving both the CNS and PNS, assuming that any other potential cause has been excluded [8]. Table 1 summarizes the classification of NPSLE. NPSLE manifestations present along a broad spectrum of clinical severity. While some symptoms, such as certain mood disorders and headaches, often present with mild-to-moderate severity, others, including cerebrovascular events, transverse myelitis, and status epilepticus, are typically associated with severe, life-threatening complications [15]. However, the intensity of any single manifestation, such as mood disorders or seizures, can vary considerably among individual patients. Cognitive dysfunction, which is one of the most prevalent features, affects approximately 38% of patients. On the other hand, the severe neurological complications like status epilepticus and transverse myelitis occur with a prevalence of only 1-2% [16]. Furthermore, NPSLE clinical polymorphism ranges from 6% to 91%, thus contributing to the wide variability in reported prevalence, and this depends on the population studied and the diagnostic criteria used [10].

**Table 1:** Different classifications of NPSLE NPSLE: neuropsychiatric systemic lupus erythematosus; CNS: central nervous system; PNS: peripheral nervous system; SLE: systemic lupus erythematosus

Classifications of NPSLE		
By anatomical location	CNS	Aseptic meningitis, cerebrovascular disease, demyelinating syndrome, headache, movement disorder, seizures, myelopathy, acute confusional state, anxiety disorder, cognitive dysfunction, mood disorder, psychosis
	PNS	Guillain-Barré syndrome, autonomic neuropathy, mononeuropathy, myasthenia gravis, cranial neuropathy, plexopathy, polyneuropathy
By clinical pattern	Focal	Cerebrovascular disease, demyelinating syndrome, movement disorder, myelopathy, seizure disorder
	Diffuse	Acute confusional state, anxiety disorder, aseptic meningitis, cognitive dysfunction, headache, mood disorder, psychosis
By pathophysiology	Inflammatory	Psychosis, seizure disorder, cognitive dysfunction, acute confusional state, myelopathy, aseptic meningitis, optic neuritis, transverse myelitis, peripheral neuropathy
	Ischemic	Cerebrovascular disease, focal seizures, mononeuropathy, vascular myelopathy, movement disorders, cognitive dysfunction
By etiology	Primary (directly attributed to SLE)	SLE-related inflammation or vascular events within CNS/PNS
	Secondary (indirect)	Infections, medication side effects (e.g., steroid-induced psychosis), metabolic disturbances (e.g., uremic encephalopathy)

Diagnosis

Diagnosis is challenging as there is an overlap with other neurological/psychiatric disorders along with the heterogeneous nature of NPSLE syndromes. Therefore, a multidisciplinary approach that involves rheumatologists, neurologists, and psychiatrists is needed. A comprehensive neurological examination is required as an initial step to guide diagnosis and differentiate NPSLE from non-SLE-related causes [17]. The cornerstone of the diagnostic workup for patients with SLE presenting with neurological symptoms is clinical attribution, determining whether an event is directly attributable to SLE after rigorously excluding competing causes. Rather than relying solely on a combination of investigations, clinicians utilize attribution frameworks that assess the temporal relationship between the neuropsychiatric event and SLE onset, evaluate competing non-SLE factors, and analyze syndrome-specific characteristics. Once alternative etiologies are excluded, neuroimaging, electrophysiological studies, and serological testing for autoantibodies are then incorporated to build a comprehensive, syndrome-specific diagnostic framework [6]. 

Neuroimaging

Neuroimaging evaluates NPSLE with ongoing advances offering deeper insight into disease mechanisms and potential diagnostic tools [18]. The conventional MRI, which is usually the first-line imaging modality, is yielding nonspecific findings that are often indistinguishable from those in SLE patients without neuropsychiatric manifestations. Currently, it is useful for ruling out differential diagnoses such as multiple sclerosis, malignancy, infarction, or subarachnoid hemorrhage [15,18]. Different types of MRI reveal different findings. For instance, the functional MRI (fMRI), including resting-state fMRI, shows altered functional connectivity and reduced nodal efficiency in regions like the insula, putamen, transverse temporal gyrus, and amygdala, correlating positively with cognitive function. Task-based fMRI shows broader recruitment of neural regions during cognitive tasks in NPSLE patients but lacks reliability in distinguishing NPSLE from non-NPSLE cases. Diffusion tensor imaging (DTI) can identify microstructural white matter damage that is unrelated to peripheral inflammation but cannot properly differentiate between NPSLE and non-NPSLE. Dynamic contrast-enhanced MRI (DCE-MRI) demonstrates increased BBB permeability, specifically in the hippocampus that is vulnerable to neurotoxic antibodies, but its diagnostic role is still being defined [18-20].

CT is also used but is limited in that it primarily helps exclude other acute causes [18-21]. PET and single photon emission computed tomography (SPECT) are two additional medical imaging techniques commonly used to measure metabolic processes in the body and perfusion abnormalities to correlate with cognitive dysfunction, but they are not specific to SLE nor to NPSLE [18,22]. Magnetization transfer imaging (MTI) shows microscopic structural changes that are not detected on conventional MRI, and magnetic resonance spectroscopy (MRS) can reveal reductions in N-acetylaspartate (NAA) and elevations in choline and lactate and thus indicate neuroinflammation and ischemia [23]. These modalities show great potential for use in NPSLE diagnosis. 

While not established for routine clinical diagnosis due to its limited specificity, the use of fluorine-18-fluorodeoxyglucose (FDG)-PET to identify regional hypometabolism in frontal, temporal, and parietal regions is currently being explored in research settings as a potential neuroimaging correlate of cognitive impairment [24]. On the other hand, arterial spin labeling (ASL), which can assess cerebral perfusion, shows limited diagnostic utility in differentiating NPSLE [20,25]. Vessel wall imaging holds promise for detecting early vascular changes, though its clinical role remains investigational [26].

Collectively, these novel imaging modalities, including DTI, MTI, MRS, ASL, and nuclear medicine SPECT/PET, demonstrate overlapping findings across studies. They highlight changes in brain microstructure, perfusion, metabolism, and functional connectivity [18]. The use of these advanced techniques in research to characterize individual neuropsychiatric syndromes within SLE, rather than treating NPSLE as a single entity due to the heterogeneity of underlying pathophysiological mechanisms, should be further explored. Their utility in tracking disease progression and monitoring response to targeted therapies should also be investigated to pave the way for personalized imaging strategies in NPSLE.

Autoantibody profiling

Numerous autoantibodies have been reported that can be helpful as diagnostic tools to assess NPSLE along with clinical disease activity. aPL, including anticardiolipin (aCL), lupus anticoagulant (LAC), and β2-glycoprotein I antibodies, are among the most well-established ones that are associated with focal thromboembolic manifestations such as cerebrovascular disease and seizures [27]. Serum aPL positivity confers a twofold increased risk (pooled OR: 2.08) of developing NPSLE compared to aPL-negative SLE patients as shown in a meta-analysis [28], while anti-Rib-P antibodies have emerged as notable phenotype-specific markers associated with psychiatric manifestations rather than NPSLE as a whole. Specifically, the presence of these antibodies is linked to a higher likelihood of experiencing psychosis and depression, with pooled odds ratios of 3.08 and 3.03, respectively, compared to patients without anti-Rib-P antibodies [29]. These antibodies are also associated with severe neuropsychiatric symptoms (three- to fourfold increased risk) and have high specificity to diagnose SLE [28,29]. On the other hand, anti-Smith (anti-Sm) antibodies, which are linked to acute confusional states, BBB disruption, seizures, and psychosis, anti-Sjögren's syndrome-related antigen A (anti-SSA/Ro) antibodies, and anti-NMDA receptor (anti-NMDAR) antibodies that are brain-specific and associated with cognitive dysfunction and mood disturbances show variable associations [30,31]. A meta-analysis suggests that the anti-NMDAR antibodies, particularly those targeting the NR2A/NR2B subunits, showed potential diagnostic relevance in NPSLE versus non-NPSLE. However, these measurements require additional validation [32].

Other antibodies that may be evaluated include aquaporin-4 (AQP4) antibodies; however, it is crucial to note that AQP4-IgG positivity in an SLE patient presenting with optic neuritis or myelitis strongly suggests a coexisting neuromyelitis optica spectrum disorder rather than serving as a direct diagnostic biomarker for NPSLE. Additional brain-specific antibodies, such as anti-triose phosphate isomerase (anti-TPI), anti-MAP-2, anti-ganglioside antibodies (AGA), and anti-neurofilament antibodies (ANFA), have also been associated with various diffuse and focal symptoms, though primarily in investigational contexts [27].

Conversely, antinuclear antibodies (ANA) and anti-double-stranded DNA (anti-dsDNA) are considered nonspecific for NPSLE [27,30]. More novel biomarkers such as anti-ubiquitin C-terminal hydrolase-L1 (anti-UCH-L1) reflect autoimmune injury to neural tissue and correlate with disease severity and higher SLE activity. The anti-glyceraldehyde-3-phosphate dehydrogenase (anti-GAPDH), anti-glial fibrillary acidic protein (anti-GFAP), BC200 RNA-specific, anti-suprabasin (anti-SBSN), and anti-MAP-2 that are elevated in NPSLE and decrease after treatment are still under investigation to confirm their diagnostic roles in NPSLE [27,30,33]. Overall, the anti-NR2A/2B, anti-MAP-2, and anti-Rib-P are among the most promising and extensively studied investigational biomarkers for NPSLE [27,34].

CSF biomarkers in NPSLE

Abnormalities in CSF such as pleocytosis and elevated protein levels are commonly seen in NPSLE [35,36]. However, emerging biomarkers, such as the CSF α-Klotho, IL-6, and lipocalin-2 (LCN2), are showing greater promise for NPSLE diagnosis and monitoring [37]. Each marker has demonstrated potential ability to distinguish NPSLE from controls in initial cohorts.

Osteopontin (OPN) has also been studied showing high specificity (reported up to 100% in preliminary studies), and its level decreases after treatment indicating potential for both diagnosis and treatment monitoring [27,38]. Furthermore, the glial fibrillary acidic protein (GFAP) levels in CSF were shown to be three times higher in NPSLE compared to non-NPSLE, with a sensitivity of 48% and a specificity of 87%, and these levels decrease following treatment [39].

The neurofilament light chain (NfL) that acts as a marker of neuronal injury has been investigated in NPSLE where its CSF levels correlate with cognitive impairment observed in NPSLE and its level declines post-treatment. Additional CSF markers such as IL-8, IFN-α, and IFN-γ can be assessed to help differentiate NPSLE from other neurological conditions; however, they are less specific [27].

Other markers such as MMP9, S100B, and TNF-like weak inducer of apoptosis (TWEAK) have been linked to NPSLE activity or MRI abnormalities, but their diagnostic performance varies between studies [27]. Recent proteomic analyses identified five differentially expressed proteins (DEPs) in CSF. Together, this five-protein panel, notably including CST6, KLK5, and TCN2, forms a diagnostic model effectively distinguishing NPSLE from non-NPSLE patients, with strong correlation to disease activity [40,41].

Among the above, CSF lipocalin-2 stands out as a promising exploratory candidate biomarker. Several candidate CSF biomarkers, especially α-Klotho, IL-6, and LCN2, show potential, but further validation in larger cohorts is needed before clinical implementation. Many of these CSF biomarkers highlight molecular pathways involved in NPSLE pathogenesis [37].

Serum biomarkers and genetic/epigenetic markers

Serum levels of neopterin, IL-6, IFN-γ, S100B, NfL, and high-mobility group box 1 (HMGB1) were shown to be elevated in NPSLE compared to non-NPSLE. These are generally more useful for monitoring disease activity than for diagnosis [27,37].

A 2024 study demonstrated that blood NfL and GFAP concentrations are higher in active major NPSLE compared to SLE without neuropsychiatric involvement, with blood NfL showing excellent sensitivity in distinguishing NPSLE. Notably, low blood NfL levels may help exclude NPSLE or other similar conditions [42].

Elevated serum vascular cell adhesion molecule-1 (VCAM-1) levels, which are usually associated with aPL, may exacerbate BBB damage in NPSLE and may have a valuable role as a biomarker [43].

Genetically, the SNP rs11797 in the three prime repair exonuclease 1 (TREX1) gene is more frequent in NPSLE patients than in those without neuropsychiatric symptoms [37,44]. Epigenetically, microRNA profiling is emerging as a powerful tool for the differential diagnosis of neuroimmune diseases. For example, while markers like miR-155 are being investigated in SLE, miR-23a (AUC 0.95-0.98; p<0.001) has demonstrated a strong ability to discriminate between overlapping conditions like neuromyelitis optica spectrum disorder and multiple sclerosis [45]. This highlights the broader potential of epigenetic markers to eventually help differentiate NPSLE from other complex neurological mimics.

A study by Stepaniuk and Shevchuk showed that elevated serum levels of soluble endoglin are significantly associated with worsening mental health, increased rates of anxiety, depression, cognitive disorders, and insomnia; the higher the endoglin levels, the higher the correlation with more frequent CNS and PNS lesions. Among the patients with endoglin levels less than 2.55 ng/ml, 29.2% had CNS and 16.7% had PNS involvement, while if the levels were higher than 4.24 ng/ml, 95.8% had CNS and 50% had PNS involvement. These findings suggest a potential role for endoglin as a biomarker in mental and neurological health, though its diagnostic utility requires further investigation [46].

The complexity and heterogeneity of NPSLE require a multimodal diagnostic approach that integrates serology, clinical assessment, and neuroimaging for accurate diagnosis and management. Additionally, it is crucial to associate the diagnostic biomarkers with specific neuropsychiatric symptoms and understand its underlying immune and neurobiological mechanisms to fully grasp its pathogenesis and guide targeted therapies.

Pathogenesis 

The pathogenesis of NPSLE is complex and is primarily driven by immune-mediated mechanisms, notably autoantibodies, inflammatory cytokines, and inflammatory cells, all of which contribute to neuronal injury. This is further complicated by genetic and environmental factors. 

Autoantibodies, cytokines, and inflammatory cells

At least 20 autoantibodies have been identified in NPSLE, with 11 targeting the brain and nine affecting the whole system [47]. They are grouped based on their mechanism of action into three main categories. Some act through procoagulant mechanisms leading to thrombosis and cerebral infarction such as aPL [48,49]. Others contribute to BBB disruption, including the anti-NR2 subunit of NMDA, anti-AQP4, anti-endothelial cell antibodies (AECA), anti-Nedd5, and anti-glucose-regulated protein 78 (anti-GRP78) antibodies [40,50-52]. Structural endothelial cell proteins also fall within this group due to their role in endothelial dysfunction and BBB compromise [53]. The third group exerts direct cellular or neurotoxic effects, such as anti-Rib-P, anti-SBSN, anti-UCHL-1, anti-GAPDH, anti-TPI, anti-MAP-2, anti-U1 ribonucleoprotein (anti-U1RNP), and anti-BC200 RNA antibodies. These autoantibodies directly impair neuronal viability, function, or intracellular processes, such as synaptic plasticity, cytoskeletal integrity, or RNA/protein handling, resulting in neuronal injury [15,40,54,55].

Autoantibodies such as anti-SS-A and anti-proliferating cell nuclear antigen (anti-PCNA) that are enriched in NPSLE patients may contribute to CNS injury by crossing the compromised BBB and thus promoting neuroinflammation. However, their exact pathogenic roles remain to be clarified [40,55].

In NPSLE, immune dysregulation drives CNS injury through a complex interplay of cytokines, complement activation, and inflammatory cells, disrupting the BBB and promoting neuroinflammation. T-helper cells are key contributors, secreting elevated levels of IL-2, IL-6, IL-10, IFN-γ, IL-17F, and granulocyte-macrophage colony-stimulating factor (GM-CSF), which promote inflammatory cascades and autoimmune activation. CD4+ T cells, including antigen-specific T cells that infiltrate the choroid plexus, initiate CNS inflammation and contribute to endothelial dysfunction [10,56,57].

Plasmacytoid dendritic cells amplify neuroinflammation via secretion of type I interferons, particularly IFN-α, which has been shown to cause synaptic loss and cognitive deficits in both human and murine lupus models [27]. Increased levels of IL-6, IL-8, monocyte chemoattractant protein-1 (MCP-1) (CCL2), interferon gamma-induced protein 10 (IP-10) (CXCL10), and granulocyte colony-stimulating factor (G-CSF) were found in the CSF of NPSLE patients, with IL-6 serving as a sensitive marker, especially in acute confusional states. Notably, many of these cytokines are produced locally within the CNS, indicating compartmentalized immune activation rather than systemic spillover [40,58,59].

Macrophage inflammatory protein 1-alpha (MIP-1α), a chemokine responsible for recruiting mononuclear cells, has also emerged as a biomarker of interest. Studies using partial least squares-discriminant analysis (PLS-DA) confirmed its significant association with NPSLE [60]. Xu and Li showed that blocking MIP-1α ameliorates disease in animal models, emphasizing its role in pathogenesis [61].

The TWEAK/Fn14 pathway is another significant contributor to BBB damage. TWEAK is a TNF-like cytokine that binds to the Fn14 receptor on endothelial and glial cells triggering inflammatory signaling via NF-κB, increasing BBB permeability, and promoting endothelial apoptosis. This pathway is linked to increased levels of nitric oxide synthase and pro-inflammatory cytokines in the CNS [15,62].

Furthermore, the complement system plays a pivotal role in NPSLE pathogenesis, especially through the interaction of C5a with its receptor C5aR1. On microglia and endothelial cells, this interaction triggers NF-κB activation, tight junction degradation, and cell death. Complement component 4d (C4d), C5b-9 complement deposition, and decreased tight junction proteins such as zonula occludens-1 (ZO-1) were shown to be associated with both diffuse and focal NPSLE. These complement markers differ by NPSLE subtype where the anti-C1q and C3/AP50 are often linked to diffuse forms, while C4 levels are associated with focal events like stroke [58,62,63].

Guan et al. suggested that the protein S100A8/A9 may play a significant role in the neuropsychiatric pathogenesis of NPSLE where its elevation in the blood was associated with fatigue and neuropsychiatric involvement, including cognitive dysfunction, in SLE patients. This protein complex is also known to contribute to inflammation via the production of neurotoxic pro-inflammatory cytokines, offering insight into mechanisms underlying neuroinflammation in NPSLE [64].

Activation of microglia further contributes to disease by promoting synaptic pruning, neurovascular unit dysfunction, and neuronal loss. Inhibiting microglia was shown to improve behavioral and cognitive symptoms in lupus-prone mouse models, underscoring their pathological role [52,56,62,65]. However, the evidence supporting these interventions remains strictly preclinical, with a notable lack of randomized controlled trials (RCTs) directly targeting microglial pathways in human subjects.

In addition, Lindblom et al. identified a distinct "interferon-high" subgroup within NPSLE patients, characterized by suppressed B cells, T cells, and natural killer (NK) cells, a phenomenon not previously well-described in the literature. This finding suggests that adaptive immune suppression may coexist with ongoing innate immune activation, indicating a unique immunopathological profile. The presence of immunological exhaustion or suppression of lymphoid cells in certain CNS-SLE cases may help explain why some patients show limited response to broad-spectrum immunosuppressive therapies, underscoring the need for more targeted treatment approaches [66].

Together, these inflammatory mediators, cytokines, T cells, dendritic cells, complement components, and microglia, compromise the BBB and perpetuate a cycle of immune-mediated neuronal injury that defines the immunopathogenesis of NPSLE.

Genetic factors

Genetic predisposition plays a crucial yet complex role in the development of both SLE and NPSLE. Several susceptibility genes have been identified, including FcγRIIIa, FcγRIIIb, integrin alpha M (ITGAM), and notably TREX1, which encodes the enzyme DNase III that is responsible for DNA degradation. Mutations in TREX1 are particularly linked to neurological symptoms such as seizures [40,67-69]. Additional genetic variants, like polymorphisms in HLA-DRB1*04/13 and single nucleotide polymorphisms (SNPs) in signal transducer and activator of transcription 4 (STAT4), are associated with an increased risk of stroke in SLE patients, independent of traditional cardiovascular risk factors or aPL status [40,54]. Moreover, the combined effect of multiple risk variants across genes such as human leukocyte antigen DRB1 (HLA-DRB1), interferon regulatory factor 5 (IRF5), STAT4, BLK, TNFAIP3, TNIP1, FCGR2B, and TNFSF13 can significantly increase the likelihood of neurological symptoms in some populations [70].

Environmental factors in the pathogenesis of NPSLE

Environmental factors play a significant role in initiating or exacerbating NPSLE in genetically susceptible individuals. Infections are notable for their ability to activate the immune system through mechanisms such as molecular mimicry, epitope spreading, and polyclonal activation, thereby promoting autoimmune responses. Pathogens like Epstein-Barr virus (EBV), *Mycobacterium tuberculosis*, and *Cryptococcus neoformans* have been implicated, with CNS infections, although less frequent, contributing significantly to morbidity [15,71,72].

Ultraviolet (UV) radiation, particularly UV-B, which induces DNA damage and apoptosis, leads to increased exposure of nuclear antigens, thus stimulating autoimmunity. UV-B also modulates immune responses by altering cytokine production and suppressing cell-mediated immunity, thus favoring B-cell activation and systemic flares. UV-A radiation penetrates deeper skin layers and may directly trigger systemic disease activity, including neuropsychiatric symptoms, although some wavelengths of UV-A may have protective effects. Sunlight exposure has been associated anecdotally with NPSLE flare-ups [73,74]. 

Drugs and toxins are recognized environmental triggers that can induce systemic autoimmunity. Various medications, such as hydralazine, procainamide, TNF-α inhibitors, terbinafine, and proton-pump inhibitors, have been linked to drug-induced lupus erythematosus (DILE); however, unlike in idiopathic SLE, severe neuropsychiatric manifestations remain rare in DILE [40].

Other environmental contributors include stress that precipitates disease flares and worsens psychiatric symptoms and smoking that is known to exacerbate systemic inflammation, thus increasing NPSLE risks [74].

Treatment

Treatment of NPSLE follows a multimodal and individualized approach, combining symptomatic management with targeted therapy for both inflammatory and ischemic mechanisms. The symptomatic treatment requires standard psychiatric follow-ups and depends on the specific neuropsychiatric presentation. Antiepileptics, antipsychotics, antidepressants, anxiolytics, mood stabilizers, and neurotropic agents, for PNS involvement, may be prescribed [15].

For inflammatory NPSLE, glucocorticoids are the first-line treatment during acute phases, with the route, dose, and duration highly individualized to the specific neuropsychiatric manifestation [21]. In severe cases, cyclophosphamide can be used in combination with glucocorticoids with superior efficacy compared to steroids alone [75]. Immunosuppressive agents such as azathioprine and mycophenolate mofetil are added to the treatment for maintenance even though their direct role in controlling neuropsychiatric symptoms is not well-defined. In refractory or severe cases, plasma exchange, intravenous immunoglobulin (IVIG), and rituximab have shown benefit, with IVIG offering additional immunomodulatory effects [27,76]. 

Biological agents, such as rituximab, also showed promise in observational studies for steroid-refractory disease. However, other agents such as belimumab and anifrolumab remain limited due to the exclusion of NPSLE patients from clinical trials [21,77]. The use of intrathecal methotrexate and dexamethasone has also improved outcomes, though they require further validation [27,78].

For ischemic NPSLE, particularly when associated with aPL, low-dose aspirin (e.g., 75-100 mg daily) for primary prevention is targeted at patients with a high-risk aPL profile or additional thrombotic risk factors. For patients with definite antiphospholipid syndrome (APS) requiring lifelong anticoagulation, warfarin remains the gold standard for secondary prevention. In general, the international normalized ratio (INR) should range between 2.0 and 3.0. Higher targets or adjunctive therapies such as low-molecular-weight heparin may be considered in recurrent events. In APS, direct oral anticoagulants are not recommended, especially in those with triple aPL positivity, due to higher recurrence rates [27,54,78,79].

To mitigate ischemic and thrombotic risks and provide potential neuroprotective effects, the management of cardiovascular risk factors and the use of statins are recommended, alongside antimalarials (e.g., hydroxychloroquine) for general systemic disease control [27,79].

Xie et al. reported a successful management of psychosis related to NPSLE. They used a combination of telitacicept, corticosteroids, and immunosuppressants, specifically mycophenolate mofetil and hydroxychloroquine, in which the patient showed sustained clinical improvement which was attributed to the synergistic immunomodulatory effects of these agents alongside antipsychotic therapy. No adverse events or infections were observed during the follow-up. This case highlights the potential therapeutic benefit of this combination for NPSLE patients with predominant psychiatric symptoms. However, further clinical studies are necessary to validate the safety and efficacy of telitacicept in broader NPSLE populations and to inform future treatment guidelines [80].

Psychotherapy and lifestyle modifications including sleep, stress reduction, exercise, and supportive care, all parts of non-pharmacological interventions, can improve quality of life and alleviate psychiatric symptoms and may aid in cognitive functioning [27].

Recent advancements in immunological profiling and translational research have opened new avenues for targeted therapies in NPSLE, based on cytokine and autoantibody phenotyping. The comprehensive assessment of multiple biomarkers may enable the development of a novel taxonomy for SLE and facilitate the implementation of personalized, immunomodulatory therapies. Machine learning-guided clustering of cytokine-autoantibody signatures has been proposed as a tool to align treatment strategies with distinct NPSLE immunotypes [60].

Recent emerging strategies such as microglia and macrophage modulation are showing promise. For instance, the use of colony-stimulating factor 1 receptor (CSF-1R) inhibitors (e.g., GW2580) effectively depleted microglia in lupus-prone mice, reducing CNS inflammation and depression-like behavior without altering peripheral autoantibody profiles [81]. Similarly, the use of sphingosine-1-phosphate (S1P) receptor modulators such as fingolimod and cenerimod improved neuropsychiatric symptoms by attenuating microglial activation, reducing lymphocytic infiltration, and preserving BBB integrity [81-83].

The BBB restoration and neuroprotection represent additional therapeutic goals. Angiotensin-converting enzyme (ACE) inhibitors such as captopril and perindopril were shown to preserve hippocampal neuronal complexity and enhance spatial memory by suppressing microglial activation in lupus models [84,85]. The TWEAK/Fn14 signaling axis also emerges as a key target where TWEAK upregulation was shown to be associated with BBB disruption and cognitive decline. Anti-TWEAK antibodies, such as BIIB023, could be repurposed for CNS lupus [86,87].

In addition, targeting IL-6, type I interferons, Th17/IL-17 pathways, and chemokines such as IP-10, MCP-1, and GM-CSF may modulate neuroinflammation and immune cell infiltration. This was shown by genetic IL-6 knockout in MRL/lpr mice leading to improved cognition and reduced astrogliosis, while IFN-α/β receptor antagonists were shown to prevent hippocampal synaptic loss. Complement inhibition, particularly via C5aR antagonists, is under investigation for ischemic neuropsychiatric phenotypes of lupus [54,88,89].

Furthermore, the remodeling of matrices and neuronal repair are also being explored. Matrix metalloproteinase (MMP) inhibitors and neurite outgrowth inhibitor A/Nogo receptor 1 (Nogo-A/NgR1) antagonists have the potential to reduce tissue damage and promote neuronal regeneration in lupus models. In addition, targeting the B-cell axis and pathogenic autoantibodies remains central to NPSLE therapy. Bruton's tyrosine kinase (BTK) inhibitors such as ibrutinib and BI-BTK-1 can reduce immune cell infiltration in the choroid plexus and improve cognitive outcomes in murine lupus models. However, although ibrutinib is approved for other indications, its repurposing for NPSLE remains hypothetical and investigational [81,89].

Neutrophil-targeted therapies are also gaining attraction given the role of NETosis and vascular inflammation in NPSLE pathogenesis. The use of inhibitors for neutrophils elastase and peptidylarginine deiminase (PAD) such as Cl-amidine was shown to reduce NET formation, autoantibody production, and thrombotic risk in lupus-prone mice. These inhibitors offered dual benefits for both inflammatory and ischemic manifestations of NPSLE [90].

Lindblom et al. highlighted the potential for personalizing NPSLE treatment by identifying patients with interferon-high versus lymphocyte-suppressed immune profiles. This stratification guided them in targeted therapies, with interferon-high patients possibly responding better to treatments like anifrolumab (anti-type I IFN receptor), C3 complement inhibitors, or calcineurin inhibitors [66]. These distinct immune signatures suggest that precision medicine approaches may improve treatment responsiveness in NPSLE.

As mentioned in the pathogenesis part, the S100A8/A9 could serve as an effective blood biomarker for identifying NPSLE-associated cognitive dysfunction in adults. Such findings provide valuable direction for developing targeted therapeutic strategies aiming at improving cognitive function in SLE patients and highlighting its potential as both a diagnostic and therapeutic target in NPSLE [64].

Figure 1 summarizes the most commonly used diagnostic methods and treatment approaches.

**Figure 1:**
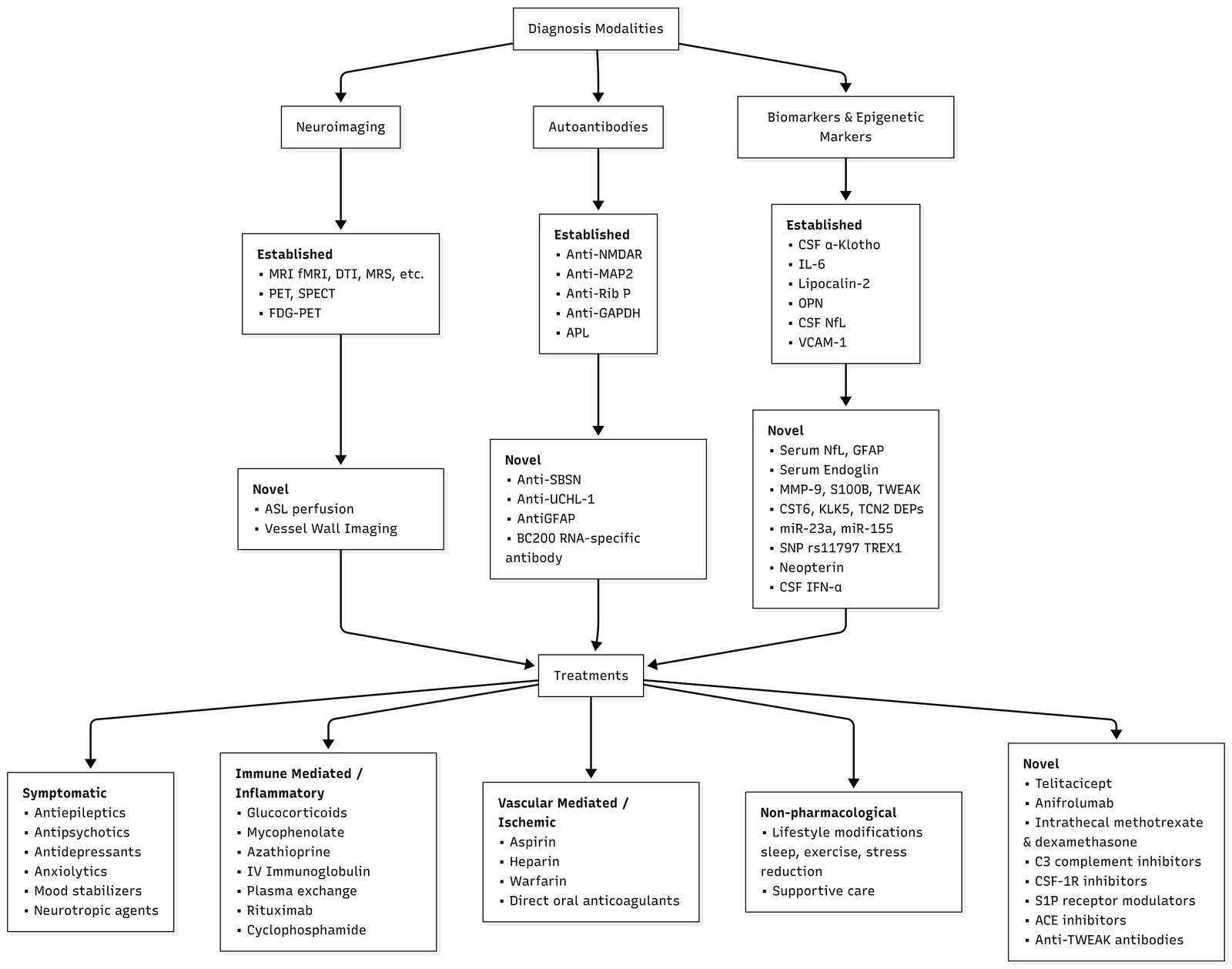
Diagnosis modalities and treatment approaches MRI: magnetic resonance imaging; fMRI: functional MRI; DTI: diffusion tensor imaging; MRS: magnetic resonance spectroscopy; PET: positron emission tomography; SPECT: single photon emission computed tomography; FDG-PET: fluorodeoxyglucose positron emission tomography; anti-NMDAR: anti-N-methyl-D-aspartate receptor; anti-MAP-2: anti-microtubule-associated protein 2; anti-Rib-P: anti-ribosomal phosphoproteins; anti-GADPH: anti-glyceraldehyde-3-phosphate dehydrogenase; APL: antiphospholipid antibody; CSF: cerebrospinal fluid; OPN: osteopontin; NfL: neurofilament light chain; VCAM-1: vascular cell adhesion molecule-1; ASL: arterial spin labeling; anti-SBSN: anti-suprabasin; GFAP: glial fibrillary acidic protein; TWEAK: TNF-like weak inducer of apoptosis; ACE: angiotensin-converting enzyme

Finally, innovative approaches such as synthetic nucleic acid-based therapies are being explored in broader contexts of neuroinflammation. In non-lupus murine models of CNS autoimmunity, nanoparticle-formulated, 1-methylpseudouridine-modified mRNA vaccines encoding disease-specific autoantigens successfully induced regulatory T cells and reduced autoreactive effector T cells without systemic immunosuppression. While this specific model does not represent lupus-related CNS disease, extrapolating this strategy to SLE presents a highly speculative, theoretical concept for future precision medicine. If successfully adapted, such platforms could hypothetically be used to selectively silence pathogenic immune responses in conditions like NPSLE [91].

To conclude, diverse studies suggested that multidisciplinary reassessment plays a crucial role in the accurate diagnosis and management of NPSLE [92]. For instance, in a study involving 304 SLE patients, 14.5% of neuropsychiatric events previously attributed to SLE were reclassified following a multidisciplinary evaluation, underscoring the potential of misdiagnosing in the absence of collaborative input [21]. Another prospective study analyzing 463 neuropsychiatric events in 293 SLE patients demonstrated that 13.8% of these events were reclassified upon multidisciplinary reassessment. Notably, 86.4% of neuropsychiatric events initially misdiagnosed as NPSLE were originally believed to have an immune-mediated origin and were treated with immunosuppressants, revealing a tendency toward over-diagnosis in clinical practice [93].

These findings highlight the challenges of attributing neuropsychiatric symptoms to SLE during initial assessments and the necessity of reevaluation. Additionally, there was acceptable interrater agreement between assessments, though diagnostic interpretations varied. The inclusion of neurology, psychiatry, and vascular medicine specialists significantly improved diagnostic accuracy, especially when reassessments were conducted after at least three months. While monitoring treatment response to immunosuppressive therapy may support ongoing clinical reassessment in initially undefined cases, this response is nonspecific. Therefore, the final diagnosis must remain based on careful clinical attribution and the rigorous exclusion of competing causes, rather than relying on treatment response as diagnostic confirmation. This approach not only enhances diagnostic precision but also impacts the understanding of NPSLE pathophysiology and informs therapeutic strategies [92,93].

Challenges and future directions in NPSLE care

NPSLE presents numerous diagnostic and therapeutic challenges, primarily due to its complex and heterogeneous nature. A major barrier to effective treatment lies in differentiating between inflammatory and vascular causes of neuropsychiatric symptoms, especially in cerebrovascular events, which may involve overlapping mechanisms. 

Currently, most therapies for NPSLE are empirical and are largely based on data from animal models, which fail to capture the full spectrum of human disease. Despite some success with immunosuppressants and anticoagulation guided by clinical presentation and aPL status, there remains no gold standard for diagnosis or treatment. As a result, delayed or incorrect diagnoses are common, often worsening patient outcomes. Timely, multidisciplinary assessment has proven beneficial and is emphasized in practice guidelines, but widespread implementation remains inconsistent.

Looking forward, the development of NPSLE-specific biomarkers is critical. Candidates such as TWEAK, LCN2, S100B, and brain-derived neurotrophic factor (BDNF), along with cytokines like IL-6 and type I interferons, are under investigation for their diagnostic and therapeutic potential. Complement pathways, including C5aR, and neuroprotective strategies involving ACE inhibitors also show promise. Clustering and systems medicine approaches may help identify distinct NPSLE subphenotypes, enabling more personalized and targeted treatments.

Ultimately, the path to optimizing NPSLE care lies in integrating emerging biomarkers, advanced imaging, and computational models into routine practice, alongside reinforcing multidisciplinary collaboration. These advancements, combined with increased clinical awareness and better therapeutic trials, hold the key to improving prognostic accuracy and treatment efficacy in NPSLE.

## Conclusions

Several key components are implicated in the pathogenesis of NPSLE, such as genetic, immunological, and environmental factors. Immune dysregulation, caused by autoantibodies, cytokines, and inflammatory cells, is the most studied. Studies have shown that these collectively damage the BBB and cause inflammatory processes that contribute to the neurological and psychiatric symptoms. These mechanisms, among other determinants, lay the framework for diagnostic approaches in which specific biomarkers can be identified. Recently, specific biomarkers have shown initial potential in differentiating NPSLE from SLE. These advances, coupled with clinical assessments, laboratory tests, and imaging studies, help support the clinical diagnosis, as no gold-standard test currently exists. These investigational findings highlight the need for further external validation. NPSLE treatment traditionally relies on symptomatic treatment and addressing underlying pathophysiological mechanisms, incorporating antiplatelet agents and immunosuppressant agents. 

The recently studied therapeutic strategies tailor personalized targeted treatment for NPSLE patients. These strategies focus on the specific manifestation of NPSLE, the predominant pathophysiology, and the severity of the clinical presentation. This individualized approach is crucial for improving patient outcomes and is supported by observational studies rather than controlled clinical trials, which are limited in this area. By understanding and targeting the precise mechanism by which NPSLE develops, personalized prevention plans can be sought. Future research should define clear diagnostic biomarkers, as well as focus on tailoring specific therapies to different clinical presentations. Treatment advances and interdisciplinary collaboration are vital to improve outcomes and optimize patient care, especially in a disease with a high mortality rate.
